# Barriers and facilitators to screen for and address social needs in primary care practices in Maryland: a qualitative study

**DOI:** 10.3389/frhs.2024.1380589

**Published:** 2024-06-17

**Authors:** Sadaf Kazi, Claire Starling, Arianna Milicia, Bryan Buckley, Rachel Grisham, Emily Gruber, Kristen Miller, Hannah Arem

**Affiliations:** ^1^ National Center for Human Factors in Healthcare, MedStar Health Research Institute, Washington, DC, United States; ^2^ Department of Emergency Medicine, Georgetown University School of Medicine, Washington, DC, United States; ^3^ Implementation Science, Healthcare Delivery Research Program, MedStar Health Research Institute, Hyattsville, MD, United States; ^4^ National Committee for Quality Assurance, Washington, DC, United States; ^5^ Maryland Primary Care Program, Maryland Department of Health, Baltimore, MD, United States; ^6^ Department of Oncology, Georgetown University School of Medicine, Washington, DC, United States

**Keywords:** social needs screening, primary care, patient stigma, documentation, community resources, qualitative methods

## Abstract

**Background:**

Social needs screening can help modify care delivery to meet patient needs and address non-medical barriers to optimal health. However, there is a need to understand how factors that exist at multiple levels of the healthcare ecosystem influence the collection of these data in primary care settings.

**Methods:**

We conducted 20 semi-structured interviews involving healthcare providers and primary care clinic staff who represented 16 primary care practices. Interviews focused on barriers and facilitators to awareness of and assistance for patients' social needs in primary care settings in Maryland. The interviews were coded to abstract themes highlighting barriers and facilitators to conducting social needs screening. The themes were organized through an inductive approach using the socio-ecological model delineating individual-, clinic-, and system-level barriers and facilitators to identifying and addressing patients' social needs.

**Results:**

We identified several individual barriers to awareness, including patient stigma about verbalizing social needs, provider frustration at eliciting needs they were unable to address, and provider unfamiliarity with community-based resources to address social needs. Clinic-level barriers to awareness included limited appointment times and connecting patients to appropriate community-based organizations. System-level barriers to awareness included navigating documentation challenges on the electronic health record.

**Conclusions:**

Overcoming barriers to effective screening for social needs in primary care requires not only practice- and provider-level process change but also an alignment of community resources and advocacy of policies to redistribute community assets to address social needs.

## Introduction

Upstream social determinants of health (SDOH), including economic stability, education access, and the neighborhood and built environment, shape individual-level social risk factors such as unemployment, housing insecurity, and insurance status. Clinical care explains only 10%–20% of health outcomes, while the remaining 80%–90% is explained by other factors including SDOH and the downstream effects on individual circumstances (1–5). In 2019, the US National Academies of Sciences, Engineering, and Medicine (NASEM) published a report outlining five strategies aimed at improving the integration of patients’ social needs (i.e., social risk factors for which a patient wants assistance) into clinical care delivery (1). Awareness about social needs can encourage adjustments in individual patient care, assist in addressing unmet needs, and, on a larger scale, align community structures to address unmet social needs within the community and advocate for policies aimed at redistributing community assets to address these social needs (2). A socio-ecological systems approach can help illustrate factors at different levels of the system in which social needs are elicited. These include the individual provider and patient level, the clinic or healthcare facility in which care activities occur, and the larger socio-technical infrastructure that shapes healthcare delivery (6–8).

Awareness of patients’ social needs is important in primary care where providers and patients maintain ongoing relationships for preventing and managing chronic diseases and preventing unnecessary health complications or hospitalizations (9–11). However, implementation of social needs screening can be complex, even for practices or providers who recognize the importance of such programs. Previous research on this topic has highlighted many of these complexities that exist at different levels of the socio-ecological system of screening for and addressing social needs. These complexities exist across various roles within the medical system, including mixed responses by patients and clinicians about who should conduct social needs screening (12–14). Complexities also exist at the clinic level, including the frequency with which screening must be conducted, and at the larger healthcare system level, including data privacy, and how this information should interface with electronic health records (EHRs) (13–18). However, there is a need to improve our understanding of patient and provider preferences for social needs screening in primary care settings and how different levels of the socio-ecological system interact with each other to impact the process of social needs screening.

National bodies in the United States such as the Centers for Medicare and Medicaid Services have invested in large-scale programs to deliver and measure the impact of social risk factor screening through the Accountable Health Communities project, and the US Preventive Services Task Force has summarized the variation in practices across settings; however, there has been no national consensus about the ideal screening tools or delivery process (19, 20). Some professional groups have begun to tackle this issue, with social needs screening institutionalized in programs such as the Bright Futures program of the American Academy of Pediatrics, while other professional groups such as the American Academy of Family Medicine developed a screening tool to encourage screening while simultaneously suggesting more research on the impact of delivering screening and referral (21).

This study aimed to apply a socio-ecological model and use qualitative techniques to identify and understand barriers and facilitators in awareness about social needs, adjustment of practice to accommodate social needs, and assistance to address unmet needs across a diverse set of primary care practices in Maryland participating in a voluntary healthcare transformation initiative (6–8).

## Materials and methods

### Conceptual model

The socio-ecological model includes nested levels (6–8). The innermost level includes individual-level stakeholders in healthcare, including clinicians, healthcare staff such as clinic administrators and medical assistants, and patients. The individual is nested within the higher level of the clinic, comprising clinic-specific factors. The healthcare ecosystem is the largest level comprising healthcare practices and the communities they are embedded in, health information technology systems, and federal- and local-level healthcare governing bodies.

### Setting

The Maryland Primary Care Program (MDPCP) is a voluntary program created by the Centers for Medicare and Medicaid Services that aims to support healthcare transformation for eligible Maryland primary care practices (22). In 2019, the MDPCP practices could choose one of two tracks. Track 1, the entry-level track that was phased out at the end of 2023, utilizes the fee-for-service model in addition to non-claims based program payments. Track 2, or the advanced track, provides higher levels of Track 1 payments and additional compensation through hybrid non-claims based and fee-for-service comprehensive primary care payments. Beginning in 2022, practices could transition to Track 3, which builds on the care delivery and performance requirements of Track 2 with enhanced financial risk for practice payments. Also beginning in 2022, MDPCP began funding the Health Equity Advancement Resource and Transformation (HEART) payment program to support providers in addressing patients' social needs (23). The practices participating in the advanced tracks are required to screen at least some of their patients for social needs and were the focus of data collection.

### Participant characteristics

We created a purposive sampling pool of 75 primary care practices for recruitment from a list of 507 MDPCP participating practices in Maryland to balance practice characteristics by (1) electronic EHR platform, (2) county, (3) Care Transformation Organization affiliation (yes/no), and (4) Track (an MDPCP classification based on criteria about services offered to beneficiaries). We selected practices to balance these characteristics proportionately (e.g., if 10% of practices in MDPCP came from a specific county, we tried to target 10% from that county in our interview sample). From this list of 75 primary care practices, we emailed contacts provided by the Maryland Department of Health to recruit practices and conducted interviews until we reached thematic saturation. In total, we included up to two representatives from 16 practices to participate in semi-structured interviews, resulting in 24 participants (four interviews included two representatives from a single practice).

### Data collection

All methods were implemented in accordance with guidelines and regulations outlined in the Georgetown-MedStar Institutional Review Board (IRB) (Protocol 4986). The interview guide was developed by researchers with expertise in social risk factor screening, quality improvement, and implementation science and modified with feedback from subject matter experts in public health and primary care (see Supplementary Material for interview guide). The interview explored current workflows for social needs screening, including clinicians and healthcare staff involved in screening, training to support social needs screening, use of screening tools, challenges in conducting screening, screening documentation, and ideal screening workflows.

We conducted virtual, semi-structured interviews via a HIPAA-compliant platform (Microsoft Teams). Interviews were led by a researcher with a master's (CS, female) or doctorate (BB, male) training in public health and skilled in qualitative research and human subjects’ compliance. Interviews also included a notetaker for documenting field notes during the interview. Data collection began after verbal informed consent was obtained for participation and audio and video recording. Interviews lasted approximately an hour. The participants received a $75 gift card.

### Analysis

Interviews were audio and video recorded and transcribed automatically through Microsoft Teams, with quality control review by a member of the research team. Data were coded using a deductive approach using Dedoose coding software (24). A researcher with advanced training in human factors engineering (SK) developed the interview codebook using grounded theory to identify barriers and facilitators around eliciting social needs (25). Three researchers (SK, AM, HA) tested the codebook by coding the same interview independently before jointly reviewing the codes. Coding discrepancies were adjudicated through consensus, and the modified codebook was applied to five additional transcripts to confirm the codebook and establish coding reliability. The remaining interviews were divided between three researchers and coded by individual researchers. An inductive approach was used to abstract themes from participant quotes within codes and summarize key ideas. SK then organized sub-themes into the socio-ecological model and mapped them to the NASEM stages of awareness and assistance (1, 6–8).

## Results

Table 1 shows the participant demographics. Interview participants represented at least 16 counties in Maryland (*n *= 3 practices each from Baltimore, Carroll, and Worcester; *n *= 2 practices each from Allegany, Anne Arundel, Calvert, Frederick, and Montgomery; *n *= 1 practice each from Dorchester, Harford, Howard, Prince George, St. Mary, Somerset, Wicomico, and unknown). Practice sizes ranged from very small to very large (1–45 providers; mean providers = 10).

**Table 1:** Demographics of interview participants (*n* = 24).

	*N*	%
Gender		
Female	18	75
Male	6	25
Race		
African American	5	20.83
Asian Indian	4	16.63
Caucasian	10	41.66
Middle Eastern	2	8.33
More than one race	1	4.17
Hispanic ethnicity	2	8.33
Role		
Care coordinator	3	12.5
Community health worker/social worker	2	8.33
Director of population health, operations	3	12.5
Medical assistant	2	8.33
Nurse	2	8.33
Physician	5	20.83
Practice manager/supervisor	5	20.83
Other (clinical lead, health management fellow)	2	8.33
Years in role: mean, range (years)	10.23, 1–35	

**Figure 1:**
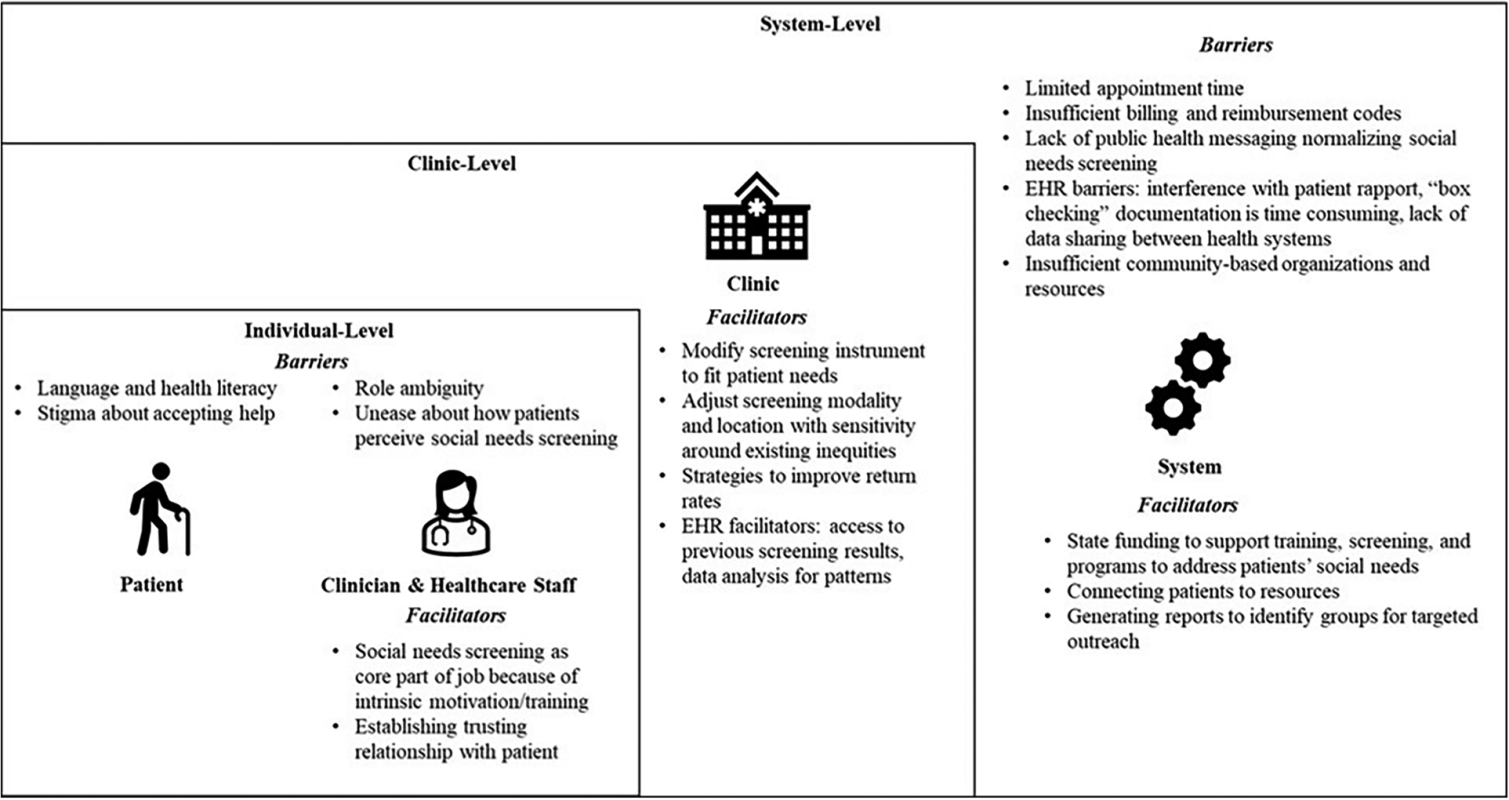
Overview of individual-, clinic-, and system-level barriers and facilitators in eliciting social needs screening in Maryland primary care practices.

Participants suggested multi-level barriers and facilitators to awareness of and assistance for social needs: (1) individual level, including clinician perceptions of patient, provider, and healthcare staff factors; (2) clinic level; and (3) system level (Figure 1).

### Individual-level factors

With regard to awareness of social needs, perceived cultural barriers were described by some providers, who noted that some patients’ cultural stigma in accepting help interfered with discussing needs in the first place. The participants were also worried about patients’ perception of social needs screening in a medical setting in terms of how data might be used and shared. Other provider barriers included discomfort in asking about social needs that they were unable to address (e.g., loneliness, patients who are victims of domestic violence and who are unwilling or unable to leave an abusive home environment). Finally, some providers were reluctant to elicit social needs because of a lack of knowledge of community-based resources to address these needs.

However, providers reported several facilitators to these individual-level factors, including addressing patient hesitancy to share social needs data through respectful and non-judgmental listening, prioritizing patient dignity, and assuring data confidentiality. In addition, intrinsic motivation led some clinicians to view asking about patients’ social needs as a core part of their job. Clinician and healthcare staff-level barriers to assisting patients with social needs include the lack of knowledge about community-based organizations (CBOs). Table 2 details the illustrative quotes on these individual-level barriers and facilitators to awareness about social needs.

**Table 2:** Illustrative quotes on individual-level barriers and facilitators that influence awareness of social needs screening in primary care.

	Barriers to awareness about social needs	Facilitators to awareness about social needs
Perception of Patient-Level Factors by Clinician/Healthcare Staff	Cultural stigma in accepting help *Participant 3:* “Finances can be sensitive to people. It's embarrassing. Who wants to say, I can't afford this? Who wants to say my electric is gonna get cut off? Nobody, and I'm not sending them money, so what am I even asking for? I mean, you have people pour their heart out to you and you can't fix it. I hate it. Adult protective service, I mean all of it.” *Participant 6 (Executive Director of Population Health): “*We have a lot of [redacted ethnicity] patients, so they don't really understand why we’re asking those questions.” *Participant 9 (Practice Manager/Supervisor):* “Sometimes it can just be fear of asking for help: ‘I don't want someone to think that I’m this type of person if I’m asking for this type of help.”	Prioritizing non-judgmental listening, respect, confidentiality *Participant 6 (Executive Director of Population Health):* “We have to earn the (patients) trust in a way to continue with that relationship and ask them these questions in a way which will ensure that we maintain that respect, the dignity of a person and, most importantly, assure them that this is not going to IRS (Internal Revenue Services) or some other authority.”
Clinician/healthcare staff-level factors	Clinician discomfort in eliciting social needs *Participant 3 (Nurse):* “I hate asking uncomfortable questions that I can't fix. So, the domestic abuse one – I hate asking it because guess who can't sleep that night? Me. And they have the right to stay with their husband who's been abusive for 50 years. They're not gonna leave now.”	Intrinsic motivation in helping address social needs *Participant 14 (Nurse):* “Honestly, I don't even know that my practice needs incentives. They are very engaged in patient care. It's like they have a bunch of children. They know their patients. And my providers will reach out and say, “Can you help with this?” I don't think they need to be incentivized to do anything for their patients. I just do what I’m supposed to do to help the patients to get things that they need. It's my job.”

### Clinic-level factors

At the clinic level, participants suggested several barriers to conducting screening including relative priorities, insufficient time to address social needs, lack of community resources or knowledge of resources, and billing structures not supporting the time spent on screening.

Participants also mentioned several clinic-level facilitators. Many of them recognized the influence of screening modality (e.g., paper, tablet), location (i.e., in the patient's home or clinic), and existing inequities (e.g., lack of access to a phone) on responses. Some therefore chose unobtrusive screening procedures to improve return rate (e.g., enclosing questionnaire in a self-addressed stamped envelope) and modified screening instruments to better fit patient needs.

A clinic-level barrier to providing assistance for social needs included difficulties in coordinating with CBOs; a facilitator was using on-site referrals and leveraging connections within (e.g., word of mouth) and outside the practice (e.g., using publicly available databases such as findhelp.org) to assist patients’ unmet social needs. Table 3 details the illustrative quotes on these clinic-level barriers and facilitators to awareness about social needs.

**Table 3:** Illustrative quotes on clinic-level barriers and facilitators that influence awareness of and providing assistance for social needs screening in primary care.

	Barriers	Facilitators
Awareness of Social Needs	Relative priorities in clinical care *Participant 11 (Physician):* “If they (clinical staff) don't see (social needs screening) as important, (and) it's one of many things that they’re being asked to do at a visit, it could easily fall. Not that it's not important to them, but that other things are more urgent.”	Screening to reduce the influence of existing inequities on responses *Participant 19 (Practice Manager):* “We have found that certain questions require a different modality of answering them. And we have found that patients are less likely to answer questions like this or answer correctly or answer honestly when asked over the telephone or if they’re asked the question using a tablet in the exam room. And so, we have pretty good success rate in mailing a questionnaire out, and we’re hoping we’ll have the same success rate with this questionnaire (by) sending it with a brief explanation and a self-addressed stamped return envelope.” *Participant 15 (Clinical Lead):* “We’ve narrowed down the social needs questions because you can probably imagine speaking to someone over 65 asking more than let's say five (questions). It becomes really difficult; they sort of lose attention and they become annoyed.”
	Appropriateness of social needs screening in a medical setting *Participant 19 (Practice Manager):* “I would suggest (to) you that the time of care is not the time to ask these questions. First of all, there's not enough time. And second, the (clinical) environment might be offensive to some patients. So, they have to be done outside (the clinic), but in a supportive way for the clinical team.”	
	Screening is seen as outside of the clinician’s role *Participant 11 (Physician):* “And this is still seen as really outside of the physician's role. Like, the physician isn't going to fix your transportation barrier. So should I be screening for this?”	
Assistance to address social needs	Lack of knowledge about community-based resources *Participant 9 (Practice Supervisor):* “Asking the question of the patient and then not being able to help them, like, you know, if we ask all these questions then we need to have the resources to help them.”	
	Challenges in coordinating with community-based organizations *Participant 10 (Community Health Worker):* “Unless there (were) agencies here in our area that are on board with the referral process if we sent them an electronic referral and we were all on the same page as far as them getting the referral, the follow through and, and things like that. I think it would work perfect, but we'd have to get everyone involved in the community.”	On-site referrals *Participant 9 (Clinic Supervisor):* “We have more success with our patients when we get them while they're in the office. We have this great care management team that we can refer to, but they're at the hospital. So, the patient goes home, and it might be a day or two before somebody calls them. If we have those resources in the office and we can go, “Hey, here's Mrs. So-and-so she has a need for transportation.” And they actually sit down and speak with someone right then and there. There's a huge difference between success rates when we get that stuff in the office.”

### System-level factors

Participants expressed several barriers related to the larger healthcare system that adversely impacted social needs screening in primary care settings. One clinician suggested that the lack of public health messaging about the impact of social needs on health contributed to the stigma of eliciting or expressing social needs in medical settings and that public health campaigns were needed to normalize the discussion of social needs as part of medical care to reduce stigma.

Several interviewees emphasized EHR-related barriers. First, EHR documentation during the clinical encounter or transferring results from paper screeners into the EHR was time-consuming. Second, EHR documentation interfered with establishing rapport, detracted from interactions with the patient, and was perceived to be unnecessary “box checking.” Third, the lack of data sharing between health systems and CBOs interfered with obtaining a complete picture of patient needs, whether social or medical. Fourth, even within a single healthcare system where providers used the same EHR, it was difficult to integrate disparate pieces of data that were scattered across the EHR to determine how to track screening for the patients’ social needs.

However, the participants also mentioned EHR facilitators. First, some EHRs enabled providers to access screening completed by other specialists, thereby reducing patient frustration with repeated screening. Second, it allowed practices to analyze data patterns to enable targeted screening and offer services to patients who met target criteria (e.g., patients aged over 65 years in a specific zip code). An additional facilitator that increased awareness of social needs was the HEART reimbursement program that funded social needs screening processes and provider training.

Participants also mentioned system-level barriers to providing assistance for social needs. These included insufficient capacity to address some social needs such as loneliness and resource lists that were difficult to navigate and access; a facilitator was using HEART funds to address patients’ needs or build practice-level programs. Table 4 details the illustrative quotes on these system-level barriers and facilitators to awareness about social needs.

**Table 4:** Illustrative quotes on system-level barriers and facilitators that influence awareness of and providing assistance for social needs screening in primary care.

	Barriers to awareness about social needs	Facilitators to awareness about social needs
Awareness of social needs	Lack of public health messaging to normalize social needs elicitation *Participant 6 (Executive Director of Population Health):* “My first part is what advocacy are we doing? Social needs issues (are) coming to the providers and the health system, but on the public health (side), what are we doing? Have we created that awareness that people understand and anticipate that this is good for them? That they should be comfortable talking to their doctors about their food insecurity issues, food safety issues and whatnot? They’ve never seen the doctor in that light. State and federal governments will have to work harder to help them understand that when you go to your doctor, please fill in this questionnaire.”	State funds to support social needs screening and training *Participant 2 (Director of Population Health Management):* “We had meetings where everyone (in the practice was) involved: the providers, MAs, registrars. And there was a presentation on what the HEART program is and how the social needs screening workflow would go.”
	EHR barriers: time-consuming documentation, non-value-added “box checking,” lack of data sharing, difficulties in data integration *Participant 12 (Physician):* “And, to be honest with you, I'm gonna speak for all the doctors and nurses and PAs and all that out there, but we're doing 500 different things. And we keep adding stuff. Somebody's gonna die for every (EHR documentation) field that we create.” *Participant 19 (Practice Manager):* “How do you integrate that information into the flow in that who within the clinical team then says, “Hey, Mr. Smith might end up in several of our cohorts. He might be diabetic; he might be a frequent flyer to the ED. He might be getting a high likelihood of clinical event score.” How do you put all of those siloed pieces of information together to provide Mr. Smith with the best care?”	EHR facilitators: access previously completed screening results, advanced data analytics to offer services to targeted groups *Participant 19 (Practice Manager):* “We have noticed that we have a lot of our HEART recipients coming from one zip code. Over the last couple of days, surveys have been sent out to all of our patients in that zip code, asking them questions about social needs, like transportation, ability to pay for their prescriptions. Do they have an advocate for their health needs? Do they have enough to eat? Are they able to pay for their household commodities like gas and electric oil, water, etc. Prior to just probably the last month or so, it had been just by word of mouth. Now we are proactively trying to create a database of those patients who are in need.”
Assistance to address social needs	Insufficient capacity of community-based organizations to address some social needs *Participant 11:* “Care management can't, uh, can't really support loneliness, right? Like we can try to connect you with social resources, but especially with the pandemic, like not all seniors with immunocompromising conditions are gonna wanna go to a senior center or the library or whatever else. Um, and there's a lot of, uh, that is a very hard social need to address and probably the one that I most frequently feel as a provider on the front line that I, I can't do a whole lot about. Um, and so it can be hard to screen for something that you feel like you can't impact.”	Using state funds to address patients’ social needs or build practice-level programs (e.g., on-site referrals) *Participant 20 (Care Coordinator):* “We've used (Aunt Bertha/FindHelp.org). From the MDPCP side, they give us a list of some resources. Meals on Wheels is one of the biggest resources I use. I just Google a lot. I don't really have special ones that I use.”
	Resource lists are difficult to navigate and access *Participant 9 (Clinic Supervisor):* “Those lists seem to be very cumbersome. There'll be 15 places listed on there and some of ‘em may not be where (patients) can get to them.”	Increasing awareness about community resources *Participant 10 (Community Health Worker):* “I'm their first and only community health worker so far, but we've grown from like three care managers to six now in two years. So I think the providers are more aware of that these are the people that can help me with these patients.”

## Discussion

Research exploring provider perceptions and acceptance of social needs screening highlights the complex operational issues that must be considered before large-scale implementation of social needs screening. NASEM recommends five strategies to improve the integration of patients’ social needs into healthcare delivery: awareness, adjustment, assistance, alignment, and advocacy (1). There is no gold standard screening tool recommended by the US Preventive Services Task Force or professional societies for primary or specialty care, introducing significant variation in whether screening is standardized and part of routine care. Our study highlights not only how barriers and facilitators to social needs screening at the individual, clinic, and system levels affect awareness about patients’ social needs but also how difficulties in addressing the strategy of providing assistance for social needs preclude this awareness.

Our findings of provider-level barriers such as perceptions of the limited utility of asking patients about social needs in a medical setting have been previously reported (16, 17, 26, 27). At the same time, we also found a limited understanding of how care activities can be adjusted to accommodate patients’ social needs. This suggests that rather than consider a linear implementation of the five NASEM strategies to improve the integration of social needs in care delivery, it may be worthwhile considering how strategies that are likely to have a tangible and direct impact on patients, such as providing assistance to patients for their social needs, may strongly impact the likelihood of engaging in awareness activities. In addition, from the perspective of healthcare providers who directly interface with patients, the ability to provide assistance may be seen as more important for direct patient care compared to strategies that boost social needs integration at larger levels of the system, i.e., alignment and advocacy. In addition, while we frame results through the socio-ecological model, these different levels are inherently connected and should be considered together in any workflow modifications or implementation recommendations. For example, some individual-level barriers by providers in conducting screening were attributable to higher-level barriers, including limited time during a typical appointment to sufficiently address patients’ social needs, balancing patient interaction with onerous EHR documentation requirements, and a relative paucity of community-based resources to address social needs.

Previous research has also found that stigma may prevent patients from sharing social needs with providers (13, 28). Our study extends these findings to suggest other multi-level strategies to combat stigma about expressing social needs in clinical settings. These include respectful and non-judgmental listening by clinicians and healthcare staff eliciting social needs, assuring patients about data confidentiality, and at a broader system level, public health messaging around social needs screening to potentially normalize such discussions in medical settings.

Research demonstrates that providers perceive many benefits of social needs screening, including tailoring care to patients’ social needs, informing community action, and building a robust referral network (16, 26, 29). Our findings were similar in that most healthcare clinicians acknowledged the importance of assessing social needs. However, clinicians also recognized several barriers that did not support this process in primary care. System-level barriers such as limited appointment times and competing priorities, insufficiently trained staff to administer the screening and coordinate referrals, and insufficient knowledge about resources to address social needs have also been raised in previous research (27, 29–31). A previous study summarizing professional medical association policy statements on social needs screening underscored this problem, highlighting how pediatrics, family medicine, and obstetrics and gynecology are leaders in encouraging screening, but do not have detailed recommendations around how and when screening should occur (32).

Our study also found that engaging in conversations about patients’ social needs and addressing needs calls for special competencies that require time and effort. Providers must navigate uncomfortable situations to elicit social needs. They must also be knowledgeable about community-based resources that exist to address social needs and know how to modify the patient's plan of care to ensure treatment access within the context of unmet needs. These tasks are important but can add to the provider’s workload if there is an expectation of also completing other clinical responsibilities within the same appointment in which social needs are assessed or addressed. Healthcare policies guiding workforce development must be reimagined to accommodate social needs and screening-related tasks to minimize provider burnout from having to perform these tasks (33).

Of note, although participants reported using HEART payment funds to support training for social needs screening, they perceived limited support in being able to effectively use these funds to assist patients’ social needs. Many providers mentioned the lack of awareness of resources within the community or how to make referrals to these resources. Therefore, they were hesitant about surfacing social needs that they were unable to address. This highlights that along with investing in improving understanding of patients’ social needs in clinical settings, there is a need to simultaneously increase investment in CBOs that can meet patients’ social needs.

In consideration of the NASEM framework, it is important that providers not only become aware of patients’ social needs and adjust the care plan accordingly but also support awareness and adjustment by ensuring that there is alignment of community resources to address unmet needs through advocacy. Thus, social needs screening implementation in primary care must consider how awareness of social needs is influenced by activities that occur at later stages in the NASEM framework, i.e., the ability to provide assistance by aligning the availability of community-based resources to address the identified needs. Our research demonstrates that providing assistance for unmet needs may be difficult unless there is system-level consideration of putting resources in place to directly address social needs (e.g., programs like HEART), designing tools to make these resources easy to locate, and designing workflows that improve the ease of coordinating referrals to CBOs. These findings are largely supported by the Centers for Medicare and Medicaid Services Accountable Health Communities Act study which found that it was challenging to resolve individual patient's social needs, even with navigators (34).

Our study had several limitations. We relied on individuals who volunteered to participate within the primary care network, which may not capture an exhaustive list of barriers and facilitators to social needs screening in primary care practices across Maryland. Additionally, we were not able to interview patients or those experiencing the screening as part of this project. However, we did have access to all potentially eligible practices and purposively sampled to get representation across various patient characteristics. Furthermore, these findings may not be generalizable to primary care practices across Maryland that are not part of this network, practices within the network that are not interested in research, or those in other states with different incentives or supports.

## Conclusions

Overcoming barriers to social needs screening in primary care requires simultaneous changes at multiple levels of the socio-ecological system in which these activities occur because engaging in activities to become aware of social needs is informed by providers’ abilities to assist with these needs by leveraging community-based resources. Thus, although it is important to implement practice- and provider-level process changes to improve screening, these individual-level changes must also be accompanied by changes at the larger system level by aligning community resources and implementing policies to redistribute community assets to address social needs.

## Data Availability

The data that support the findings of this study are available from the corresponding author upon reasonable request.
