# Supplementary material for: Barriers and facilitators to screen for and address social needs in primary care practices in Maryland: a qualitative study
Source: Front Health Serv. 2024 Jun 17;4:1380589. doi: 10.3389/frhs.2024.1380589 (PMC11215188; doi:10.3389/frhs.2024.1380589)
Supplement: Supplementary file 1 [file Table1.docx]

**Supplementary Materials – File 1: Interview Guide**

Hi, *My name is {insert facilitator name}. I am a part of MedStar Health Research Institute. We are working to understand social risk factor screening, demographics data collection, and referral processes within the MDPCP program* *.*

*The information you share will help us understand gaps and challenges you see in identifying social risk factors and detailed demographic data and identify solutions to improve processes for clinicians, patients, and other involved individuals. We thank you in advance for your time and sharing your opinions.*

*Today's discussion is about your overall experience, rather than assessing your clinical knowledge about screening or patient assessment. Any information you provide will be kept confidential. Please feel free to share as much or as little of your experience or the experiences of others as you like. You may stop participating in this interview at any time. Any and all feedback is valuable information.*

*Any questions you had about the informed consent document we sent you? [If yes, answer, if no, continue]. This session will be audio and/or video recorded. If you are comfortable, we will start the recording now.*

**[Note for interviewer: particularly important questions in bold, if time is short focus on these]**

**Understanding social needs among patients**

1. **How do you currently become aware of your patient’s social needs, such as housing, food, a need for social support, etc.?**

Probe: Please tell me about any existing process for discussing social risk factors among your Medicare patients.

- 1. Screeners, information from social workers
  2. Who talks to patients about social risk factors?
  3. Payer-specific care management programs
  4. Probe: issues such as food insecurity, housing, transportation, social support, intimate partner violence, utilities.

1. We are hoping to understand how your practice may have different workflows depending on whether the patient is covered by Medicare. Can you tell me how questionnaires or time with patients differ for a patient who might be covered by MDPCP payments?
2. What are the most common social needs identified? (i.e., do your patients cite specific needs regularly)?
3. **What are the challenges in identifying patient social risk factors? (e.g., patient hesitant to discuss personal details/needs, time with patient, etc).**
4. *If any formal screener is discussed,* do you use a particular social risk factor screening tool in your practice (e.g., PRAPARE, ACH, internally developed)?
   1. (If Yes) Please describe the screening tool(s) that you use?
   2. What is the delivery format of the screening tool(s) (e.g., paper, electronic based)?
   3. Are there constraints/restrictions that impact the screener used? If yes, please describe.
      1. Organization / Health system
      2. Physical environment
      3. Resource availability
      4. Payment models
   4. (If maybe or I don’t know) Would you be willing to send us a copy of your screener after this interview?
5. **Would you like to learn more about advantages or disadvantages of commonly used social needs screeners and how you might find one that works well at your clinic?**
   1. [If yes]
      1. **What supports do you need to implement screening questions into your workflow?**
         1. Probes: EMR management, training in how to ask sensitive questions
      2. What questions related to social or practical needs do you think are most important to ask your patient population?
         1. Social isolation? Transportation? Affording medications?
      3. Who should conduct the screening?
      4. When should the screening be done?
   2. [If no]
      1. What are some of your concerns with using a social needs screener?
6. **If you could implement any screening process that would capture social risk factors for patients in your clinic, what would it look like?**
   1. Format (paper, electronic)
   2. Stand-alone screener versus inclusion in another screening/assessment tool
   3. All patients or only Medicare patients?
   4. What resources and/or technology would make it easier to collect and keep social needs data up to date?
   5. Are z-codes currently identified?
      1. If not, would this interest you?
7. **How are social needs documented when they are identified?**
   1. Structured fields in EMR?
   2. Free text notes?
   3. Email/phone call to social worker?
8. Who addresses identified needs?
   1. What referral process is in place?
   2. What payment models support addressing needs?
   3. Are there organizations in your community that can address those risk factors?
9. What types of supports like trainings or guides have you received in asking about patient social risk factors?

Probes:

- 1. Training in asking sensitive questions
  2. MDPCP supports
  3. CBO/NGO guidance or training

1. **What kind of resources would be helpful to you in asking about social risk factors?**

Probes:

- 1. Referral resources
  2. Training around asking sensitive questions
  3. EMR fields/prompts

1. How would you like to receive information on relevant patient social risk factor screening or referral resources?
   1. Online resources
   2. Print materials
   3. Direct email
   4. Trainings?
2. **When you find out about a patient’s social risk factors, how does it affect clinical care?**
   1. Probe: can you tell me about a time when learning about a social risk factor changed care?

**Understanding demographic data collection process**

*I am now going to move on to ask about demographic data collection. We know everyone is at a different stage of the journey. The collection of demographic data has been shown to help inform treatment plans and ensure equitable treatment outcomes. Many of our questions are to get a sense of the current state. Again, we know there are many competing demands, and everyone is at a different stage.*

1. **There is emerging evidence around the importance of detailed demographic fields. We are interested in learning whether practices collect demographics such as gender identities other than male/female, sexual orientation, pronouns, detailed race/ethnicity categories, or disabilities. Note: If they are not sure about what they collect, list out the above list**

Probe:

- 1. Are there any demographic fields that you feel are particularly limited that are collected at present?
  2. Are there any demographic fields you would like to collect or expand on that are not currently collected?

1. How are patient demographics currently documented?

Probe-

- 1. Self-administered, asked during visit
  2. Portal
  3. Paper forms

1. **What are the biggest challenges in collecting patient demographics?**
2. **How do you use the demographic data collected?**
3. What additional resources would be helpful to you in considering relevant demographic data to collect?
   1. Trainings on state-of-the-art measures
   2. EMR adaptations/fields
4. How would patient demographics be used in your practice?
   1. Aggregate, summary data?
   2. Patient-specific data?

**Resource development & technical assistance**

*Our next step is to create a toolkit for best practices in social risk factor screening and demographic data collection.*

1. **What types of tools would be most useful to you if you were considering adopting new measures?**
   1. Case-studies of how to use data in care
   2. Literature on social risk factor implementation
   3. Tracking systems
   4. Online screening tools
   5. Online referral tool (for us or the patient?)
2. We plan to offer on-site technical assistance to support some clinics considering new processes for data collection. Do you anticipate requesting on-site help? If so, what type of supports would be useful on site?
3. **How would you like to receive technical assistance to address challenges in implementation?**
   1. Probe: do you prefer print, electronic materials? synchronous vs. asynchronous? presentation of support tools? Learning health model? Interactive?
   2. Frequency?
4. What kind of incentives would encourage providers to complete trainings on demographic data collection or social risk factor screening?
5. What kind of incentives would encourage clinics to expand demographic data collection or social risk factor screening?
6. What kind of incentives would encourage clinics to report on demographic data collection or social risk factor screening?
7. **Is there anything I haven’t asked that you think I should know about social needs screening or demographic data collection at your practice?**

## Demographics

1. **What is your gender identity?**
2. **What is your race?**
3. **What is your ethnicity?**
4. **What is your role within your organization?**
5. **How many years have you been with your organization?**

*Thank you for participating in this interview. Your feedback is extremely helpful. We truly appreciate you and your time. As thank you for your time we are offering you a $75 gift card.*

*Would you prefer to receive it via email or mail? If email, should we use the email we scheduled this interview on? If by mail what address should we send it to? It will take about 2 weeks to process the gift card*

*Do you have any questions after this interview?*
