# Supplementary material for: Barriers and facilitators to screen for and address social needs in primary care practices in Maryland: a qualitative study
Source: Front Health Serv. 2024 Jun 17;4:1380589. doi: 10.3389/frhs.2024.1380589 (PMC11215188; doi:10.3389/frhs.2024.1380589)
Supplement: Supplementary file 2 [file Table2.docx]

**Supplementary Materials – File 2: Interview Codebook**

| **Code** | **Question/Description** |
| --- | --- |
| Challenges in eliciting social needs | Q4: What are the challenges in identifying patient social risk factors? (e.g., patient hesitant to discuss personal details/needs, time with patient, etc). |
| Facilitators in eliciting social needs | Any factors (e.g., workflow, technology design) that help in eliciting patients social needs |
| Resources desired to support social needs elicitation | Q11: What kind of resources would be helpful to you in asking about social risk factors? |
| Individual level barrier/facilitator | Factors relevant to individual stakeholders in the socio-ecological system (e.g., patients, providers, clinic staff) |
| Clinic-level barrier/facilitator | Factors relevant to the day-to-day operation of the healthcare clinic |
| System-level barrier/facilitator | Factors relevant to the healthcare ecosystem in which social needs screening occurs, including clinics and hospitals, communities, community-based organizations, health information technology systems, policy-making bodies |
| Awareness about patients social needs | Q1- How do you currently become aware of your patient’s social needs, such as housing, food, a need for social support, etc.? |
| Who administers screeners? how are they administered? | Provider, self-administered, medical assistant, community health worker |
| Common social needs | Q3. What are the most common social needs identified? (i.e., do your patients cite specific needs regularly)? |
| What happens if social needs are identified? | Q13: When you find out about a patient’s social risk factors, how does it affect clinical care? |
| Preferred modalities of receiving information to support social needs elicitation | Q12: How would you like to receive information on relevant patient social risk factor screening or referral resources? |
| Formal screener | Name of identified tool, or custom tool |
| Named EHR | Name of the EHR used by the facility |
| Aggregate utilization of social needs data | How are population-level data aggregated? |
| Integration of Z codes | Any mention of using or wanting to use ICD-10 Z-codes to code social needs |
